# A brief report on the development of a theoretically-grounded intervention to promote patient autonomy and self-management of physiotherapy patients: face validity and feasibility of implementation

**DOI:** 10.1186/s12913-015-0921-1

**Published:** 2015-07-05

**Authors:** James Matthews, Amanda M. Hall, Marian Hernon, Aileen Murray, Ben Jackson, Ian Taylor, John Toner, Suzanne Guerin, Chris Lonsdale, Deirdre A. Hurley

**Affiliations:** Institute for Sport and Health, School of Public Health, Physiotherapy and Population Science, University College Dublin, Dublin, Ireland; The George Institute for Global Health, Nuffield Department of Population Health, The University of Oxford, Oxford, UK; School of Sport Science, Exercise and Health, University of Western Australia, Perth, WA Australia; School of Sport, Exercise & Health Sciences, Loughborough University, Loughborough, LE11 3TU UK; Department of Sport, Health and Exercise Science, University of Hull, Cottingham Road, Hull, HU6 7RX UK; School of Psychology, University College Dublin, Belfield, Dublin, Ireland; Institute for Positive Psychology and Education Faculty of Health, Australian Catholic University, Strathfield, NSW Australia

**Keywords:** Implementation, Knowledge translation, Behaviour change, Theoretical domain framework, Physiotherapy, Self-determination theory, Low back pain

## Abstract

**Background:**

Clinical practice guidelines for the treatment of low back pain suggest the inclusion of a biopsychosocial approach in which patient self-management is prioritized. While many physiotherapists recognise the importance of evidence-based practice, there is an evidence practice gap that may in part be due to the fact that promoting self-management necessitates change in clinical behaviours. Evidence suggests that a patient’s motivation and maintenance of self-management behaviours can be positively influenced by the clinician’s use of an autonomy supportive communication style. Therefore, the aim of this study was to develop and pilot-test the feasibility of a theoretically derived implementation intervention to support physiotherapists in using an evidence-based autonomy supportive communication style in practice for promoting patient self-management in clinical practice.

**Methods:**

A systematic process was used to develop the intervention and pilot-test its feasibility in primary care physiotherapy. The development steps included focus groups to identify barriers and enablers for implementation, the theoretical domains framework to classify determinants of change, a behaviour change technique taxonomy to select appropriate intervention components, and forming a testable theoretical model. Face validity and acceptability of the intervention was pilot-tested with two physiotherapists and monitoring their communication with patients over a three-month timeframe.

**Results:**

Using the process described above, eight barriers and enablers for implementation were identified. To address these barriers and enablers, a number of intervention components were selected ranging from behaviour change techniques such as, goal-setting, self-monitoring and feedback to appropriate modes of intervention delivery (i.e. continued education meetings and audit and feedback focused coaching). Initial pilot-testing revealed the acceptability of the intervention to recipients and highlighted key areas for refinement prior to scaling up for a definitive trial.

**Conclusion:**

The development process utilised in this study ensured the intervention was theory-informed and evidence-based, with recipients signalling its relevance and benefit to their clinical practice. Future research should consider additional intervention strategies to address barriers of social support and those beyond the clinician level.

## Background

Low back pain (LBP) has recently been ranked as the leading cause of disability worldwide [1]. Within Ireland, it has been estimated that 395,000 or 11.9 % of people aged 18 years and over had a chronic back condition in 2010 [2]. While no “cure” using traditional therapeutic approaches has been identified, current clinical practice guidelines (CPGs) promote a self-management approach as best practice [3–5]. Non-pharmacological self-management strategies such as exercise [3] are based on high quality evidence derived from randomized controlled trials and represent current best practice for this condition. However, promoting self-management necessitates a change in the clinical behaviours of many healthcare professionals (HCPs) trained to use a biomedical approach [6]. For example, promoting self-management involves the clinician to act as a co-regulator in the treatment process working in partnership with the patient to take responsibility for their symptom management rather than using a traditional biomedical approach to care. Thus, a major challenge with uptake of CPGs is changing HCP behaviour to support patient autonomy to adopt a self-management approach [7].

Evidence suggests that HCPs can influence patients’ motivation and ultimately their maintenance of health-conducive behaviours through their communication style and adoption of a patient centred approach [8]. This approach based on self-determination theory (SDT) [9] proposes that autonomous motivation leads to greater persistence with the targeted behaviour and enhanced psychological wellbeing whereas controlled motivation can result in poor long term engagement with the targeted behaviour. According to SDT, autonomous motivation is characterised by self-endorsement of the behaviour and a belief in its value while controlled motivation typically relates to engaging in a behaviour due to feelings of guilt or external pressures such as coercion. The development of autonomous motivation can occur through the social environment and the autonomy supportive communication behaviour of a significant other [10]. In a health context, the concept of autonomy supportive communication behaviour represents an interpersonal climate whereby the HCP places the patient at the centre of the treatment experience, for example, taking the perspective of the patient into account, providing relevant information and opportunities for patient input and choice [10].

Recently, several interventions across different populations have supported the use of SDT derived communication behaviours by HCPs to promote a patient’s active role in the treatment process and ultimately their maintenance of health-conducive behaviours, including medication adherence [11], physical activity [12], smoking cessation [13] and dental hygiene [14]. More recently, SDT was used to develop a series of communication strategies as an intervention to improve the management of low back pain in physiotherapy settings; specifically the strategies were aimed at improving patients’ autonomous motivation to increase and maintain their physical activity levels. Full details of the 18 communication strategies used in the intervention entitled the *Communication Style and Exercise Compliance in Physiotherapy* (CONNECT) trial can be found in the study protocol [15]. Initial evidence from this trial found that physiotherapists who completed the CONNECT training provided greater autonomy support for patients’ needs compared to physiotherapists who had no training [16].

Although, there is evidence to support the effectiveness of this autonomy supportive communication style, adopting this behaviour in clinical practice may prove challenging without the addition of appropriate education or training resources. Recent Cochrane systematic reviews have recommended [a] continuing education meetings [17], [b] educational outreach visits [18], [c] local opinion leaders [19] and [d] audit and feedback [20] as effective evidence-based strategies to change professional practice. While, a multi-faceted approach to changing healthcare professional behaviour is recommended, many studies have failed to prospectively identify barriers to implementing interventions thus limiting their effectiveness. Furthermore, research of the methods used to identify barriers and tailor interventions to address them has been advocated by the Cochrane collaboration [21]. Commonly reported barriers to implementing evidence in practice are lack of appropriate skills, lack of support and time constraints [22–24]; however, specific barriers relating to the use of SDT-based communication strategies have not been identified. Consequently, there is a need to focus on identifying barriers and then developing and evaluating evidence-based interventions to support knowledge mobilization and effective implementation [25]. To support this process, tailored interventions that aim to change clinician behaviour and to support the uptake of evidence into practice are recommended [26].

The increasingly used Theoretical Domains Framework (TDF) provides a validated systematic, theoretically derived framework of 14 domains for identifying the main factors believed to enhance or impede practitioner behaviour change, for example, knowledge, skills, beliefs about capabilities, beliefs about consequences, optimism and professional identify [27], as well as facilitating the selection of evidence-based strategies to address them. Indeed, the TDF has been applied to a small number of studies examining LBP and osteoarthritis [28–30]. Therefore, the main aim of this study was to develop a pragmatic intervention for changing provider behaviour; specifically the Knowledge Exchange and Delivery Support (KEDS) intervention for changing physiotherapist behaviour relative to communication strategies with back pain patients. A secondary purpose of the study was to pilot-test our protocol for implementing the KEDS intervention within a clinical setting to obtain preliminary data in order to identify its acceptability and make any necessary refinements.

**Fig. 1:**
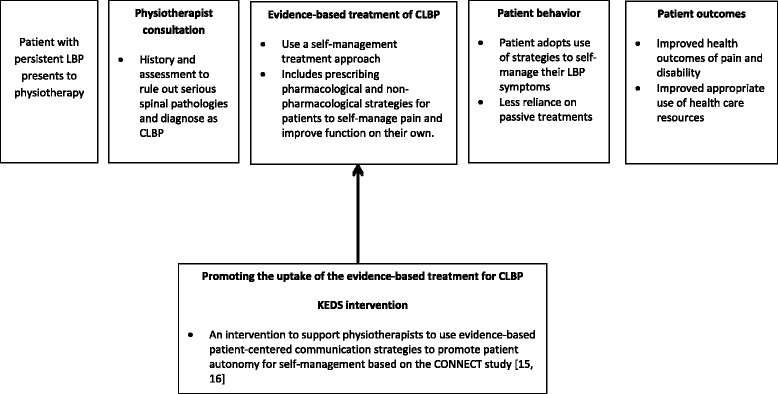
The theoretical model underpinning the rationale for developing the KEDS intervention

Figure 1. Presents the theoretical rationale underpinning the development of KEDS as an intervention to support the uptake of evidence-based communication strategies used in the CONNECT trial [15].

## Methods

This study took place within primary care clinics in the greater Dublin region providing acute and chronic care. It was approved by the appropriate Research Ethics Committee for primary care services in the greater Dublin region. All participants provided written consent prior to taking part in the study.

### Phase 1 - initial development of the KEDS intervention

To inform the development of the intervention, two focus groups were conducted with a purposive sample of primary care physiotherapists (n = 9) who had training and experience using the target behaviour in practice as part of the study entitled the CONNECT trial [15]. The TDF was used to inform the focus group interview guide in order to identify the barriers (and enablers) to implementing the SDT-based communication behaviour change. Both focus groups were led by an experienced qualitative researcher (SG) who explored the reasons why physiotherapists did/did not use particular communication strategies during individual LBP patient management. Questions were developed to specifically explore the TDF domains for the targeted behaviour. The results provided detailed information regarding key barriers and enablers to implementing the communication strategies in practice.

Specifically, eight barriers and enablers were identified across organizational, managerial/administration, physiotherapist and patient levels. These barriers and enablers were reviewed by the research team and agreement was reached as to which could be reasonably targeted within the KEDS intervention. For example, it was agreed that the barriers related to the domains, environmental context and resources (e.g. lack of resources or long waiting lists) and social/professional role and identify (at the patient level, e.g. patient expectations of passive treatment) were beyond the scope of this study. Consequently, six barriers and enablers were identified as modifiable and feasible to address within the KEDs intervention. These included physiotherapist knowledge, skills, social influence, professional role, beliefs about capabilities and behavioural regulation.

Using a combination of theory and evidence, intervention components which could address the selected barriers and enablers were then chosen by the research team. First, specific behaviour change techniques (BCTs) for each modifiable barrier and enabler were chosen using a matrix [31] which enables the mapping of BCTs to theoretical domains. Second, the mode of delivery was selected based on a review of recent evidence [e.g. Cochrane systematic reviews; 17–20], with the aim of maximising BCT implementation within the intervention. Finally, to ensure the components were likely to be feasible, acceptable and locally relevant, primary care physiotherapy managers were consulted regarding the proposed behaviour change techniques and modes of delivery. No changes to the intervention components were required based on this consultation process.

### The proposed KEDs intervention

A list of the targeted barriers and enablers and their associated evidence-informed components included in KEDs intervention can be found in Table 1. In short, these were:

**Table 1:** Description of the process used to develop the KEDS intervention

The barriers and enablers identified from the focus groups	The TDF domains linked to the barriers and enablers identified from the focus groups	Intervention components (BCTs, mode & content) selected to overcome the modifiable barriers and enhance the enablers
Physiotherapists lack awareness of certain communication strategies	Knowledge	*BCT:* Information regarding the behaviour
		*Mode:* Continuing education meeting
		*Content:* Information was presented about the specific communication strategies. For example, physiotherapists watched a video where the use of these communication strategies with a typical chronic low back patient was demonstrated
Physiotherapists are unsure of how and when to use certain communication strategies with patients	Skill	*BCT:* Goal-setting and problem-solving
		*Mode: 1.* Continuing education meeting; and
		2. Individual coaching session
		*Content:* 1. At the end of the education meeting, physiotherapists were encouraged to set a goal and develop an action plan to practice one/ two communication strategies. Physiotherapists provided with a goal and action sheet to facilitate this. 2. Physiotherapists in collaboration with the coach set goals related to implementing the communication strategies in practice and problem-solved any likely barriers to implementation.
		*BCT:* Self-monitoring
		*Mode:* Individual coaching session
		*Content:* After each audio recorded patient consultation, physiotherapists recorded and reflected on their use of the communication strategies during the consultation
Physiotherapists lack self-confidence in their ability to successfully implement certain strategies	Beliefs about capabilities	*BCT:* Goal-setting and problem-solving Mode & content as described for the Skill domain
		*BCT:* Self-monitoring Mode & content as described for the Skill domain
		*BCT:* Feedback
		*Mode*: Individual coaching session
		*Content:* Verbal and written feedback provided to each physiotherapist during the coaching session regarding their use of the communication strategies based on audio recorded patient consultations
Physiotherapists are working in isolation. There are limited social networks to encourage or support the use of new strategies	Social influences	*BCT:* Social processes of encouragement and support
		*Mode:* Continuing education meeting
		*Content:* Group based discussion within the meeting where physiotherapists shared the positive experiences they had using these communication strategies with patients and discussed ways by which they could support and encourage their colleagues in using these strategies in their clinical practice
Physiotherapists’ beliefs regarding communication being a core part of their professional role is a motivating factor to implement these strategies effectively	Professional role and identity (physiotherapist perspective)	*BCT:* Persuasive communication
		*Mode:* Continuing education meeting
		*Content:* Respected physiotherapist who is part of the research team discussed the evidence and benefits of using these communication strategies with patients to promote active management of their LBP. Group based discussion to allow opportunity to discuss importance of communication among colleagues at the meeting
Physiotherapists do make conscious and practical adjustments (e.g., reminding themselves of these strategies prior to a consultation) to their practice in order to improve their implementation of these communication strategies.	Behavioural regulation	*BCT:* Prompts, triggers, cues
		*Mode:* 1. Continuing education meeting; and 2. Individual coaching session
		*Content: 1*. Physiotherapists provided with a communication strategy reminder sheet at the end of the continued education meeting which could be placed in patients’ files to remind the physiotherapist to use these strategies in their practice. 2. Physiotherapists emailed a copy of the agreed updated goal and action sheet within 24 h of the coaching session.
*Barriers identified from the focus groups which were deemed beyond the scope of the study*		
Patients can present with a specific expectation regarding treatment and a preconceived perception of the role of a physiotherapist in the management of their condition (i.e. expectation of hands on treatment, passive role in their own treatment)	Professional role and identity (patient perspective)	
Clinics have long waiting lists, less staff, and fewer resources. These communication strategies become secondary in a time pressured environment	Environmental context and resources	

Note: Details of focus group participants: Mean age = 37.4 years (SD = 6.4); Average years total physiotherapy experience = 12.6 years (SD = 5.5); Average years’ experience in primary care clinical practice = 10.2 years (SD = 5.6)

(i) *Continuing education meeting:* The once –off education meeting was designed in line with recommendations from a Cochrane review (e.g. a facilitated group workshop, using both didactic and interactive methods) [17]. The BCTs selected for implementation in this part of the intervention included, information regarding the targeted behaviour, social process of encouragement and support, persuasive communication and goal-setting. These components aimed to target domains such as knowledge, skills, social influence, professional role and identity (physiotherapist level). This meeting was scheduled to last 2.5 h and was open to all physiotherapists within the primary care site. A registered Psychologist (JM) and chartered Physiotherapist (DH) who had experience in using these communication strategies and had published related research in peer reviewed journals in the last five years, facilitated the meeting. Broadly, the meeting began with an introduction to the theory and evidence for the SDT-based communication strategies. This was followed by group based discussions/exercises as to how these strategies could be used in practice. Finally, practical steps as to how to implement these communication strategies in practice were identified and shared by the participating physiotherapists.
(ii) *Two individual coaching sessions*: This type of coaching session was designed in line with recommendations from the literature (e.g. the process included more than one coaching session, feedback was provided both verbally and in writing and included collaborative goal setting and action planning) [20]. BCTs used with this mode of delivery included, feedback, goal setting, problem solving, and prompts and cues. These sessions were designed to target the TDF domains such as skills, beliefs about capabilities and behavioural regulation. Each session was scheduled to last one hour and was led by the same registered Psychologist who facilitated the education meeting. Additional tools used to support the coaching process were:
  - *Audio recordings* were collected by the physiotherapists at their consultations with selected chronic LBP patients for assessment and feedback. Directly after a physiotherapist-patient consultation, the audio recording was collected by a research assistant (MH) and submitted to the coach (JM) for review. The results of the review were used to inform the focus of the subsequent coaching session with the physiotherapist.
  - *Self-monitoring and reflection sheets were completed* by each physiotherapist after each audio recorded consultation during the intervention phase. The sheet asked physiotherapists to record and reflect on their use of SDT-based communication strategies during their patient consultations (e.g. what they felt they did or did not do well?). These sheets were reviewed by the coach along with the audio recordings and informed the feedback provided by the coach during the subsequent session.
  - *Goal setting and action planning* occurred at the end of each coaching session followed by an email from the coach to the physiotherapist within 24 h with a brief re-cap of the key points of the session and a copy of the agreed updated goal(s) and action sheet for the physiotherapist to refer to.

### Phase 2 - refinement of the KEDS intervention

A pilot test of the protocol to implement KEDS was undertaken with two physiotherapists at two separate primary care sites to explore face validity, feasibility of intervention delivery as well as to collect preliminary objective data on change in provider communication behaviour. Face validity and feasibility were assessed via semi-structured interviews with participating physiotherapists. Specifically, we were interested in determining if participants perceived that the KEDs intervention addressed key barriers and enablers to using communication strategies in practice in order to make refinements to the intervention components. Additionally, we assessed if participants found the KEDs intervention “acceptable”. Acceptability of the intervention was defined as the perception of the participants that the intervention is agreeable, credible and has relative advantage compared with current treatment practices [32]. It is suggested that if an intervention is acceptable it increases the likelihood of it being adopted in practice [33]. Both interviews were audio recorded, transcribed and reviewed by two members of the research team.

Preliminary data on physiotherapist communication behaviour change was also collected using the Health Care Climate Questionnaire (HCCQ) [34]. The HCCQ assesses the level of autonomy support provided by a health care practitioner to the patient through their communication behaviour. It has good reliability and validity in similar populations [34]. The 6-item version of the HCCQ was used in the present study and includes statements such as “the physiotherapist conveyed confidence in the participant’s ability to make changes”. These statements were assessed on a seven-point Likert scales anchored in 1 = not true at all, 4 = somewhat true and 7 = very true [34]. The scores are averaged for a total scale ranging from 1 to 7.

## Results and Discussion

The aim of this study was to develop an implementation intervention that could be used to promote physiotherapist behaviour change in primary care. Specifically, the intervention was developed to support physiotherapist’s enhanced use of theory-informed, evidence-based communication skills in clinical practice. Our development approach was systematic and allowed us to choose behaviour change techniques and delivery modes informed by theory and evidence that addressed some of the specific barriers and enablers identified by key stakeholders in the local context. The subsequent pilot-study with two physiotherapists, allowed us to consider how this intervention could be optimized, tailored and refined prior to a more detailed testing of effectiveness.

### Refinements

During the pilot-testing we found that, on the whole, both physiotherapists were positive regarding the success of the KEDS intervention in addressing the barriers of knowledge, skills, beliefs about capabilities and behavioural regulation; particularly, the coaching process in general and specifically the goal and action sheets and audio-recording review. For example, one physiotherapist noted in the follow-up interviews that “the one on one coaching was fantastic and I think really helped to cement all the learnings”. However, they felt the barrier of limited social support was not addressed effectively through the chosen intervention components; primarily due to wider environmental constraints, of working in isolation with few opportunities to discuss, support and encourage their peers. This is illustrated by the following physiotherapist comment, “we wouldn’t really be sharing our work practices as such, I don’t think I would have the opportunity to sit down with them (i.e. colleagues) and ask them about their experience with the communication skills”. Lastly, in terms of acceptability, both physiotherapists considered the intervention to be relevant and, beneficial to their practice and signalled a desire to continue using these skills in their daily interactions with patients. For example, one of the physiotherapists stated, “I would see myself going forward and using it (i.e. autonomy supportive communication style) the overall benefit to me is the sense of being in partnership with the patient”. Thus, as a result of these initial findings, we intend to use most of the selected strategies but would recommend enhancing the continuing education meeting to further address social support.

In addition, preliminary assessment of physiotherapist behaviour change with the HCCQ revealed an important finding. As part of promoting autonomy supportive behaviour, the KEDs intervention also addresses how to identify and reduce controlling behaviours (e.g. contingent reward or conditional acceptance) which has been negatively associated with long-term behaviour change [35]. Feedback from the semi-structured interviews reinforced the discussion and recognition of controlling behaviours as a useful part of the intervention. Unfortunately, the HCCQ only measures autonomy supportive behaviours. Therefore, based on this finding and in line with recent health related research [36], we would refine the intervention assessment to include measures of autonomy supportive *and* controlling behaviours to fully assess change in physiotherapist communication behaviour.

### Strengths

The MRC guidelines for design and evaluation of complex (e.g. behaviour change) interventions, recommend that researchers fully describe the rationale underpinning the development process and map intervention components to theory and outcome to allow for meaningful evaluation which we have done using the TDF. Thus, our study differs from many previous implementation interventions which did not rely on theory to design interventions [37] and expands on the limited number of theory-informed, tailored interventions developed using a rigorous methodological approach. Moreover, by tailoring the intervention to address specific TDF barriers/enablers and linking those with specific behaviour change techniques, we ensured a better understanding of how change within the intervention might be achieved [38] and increased the opportunity to change clinical practice.

### Recommendations for future research

This intervention only addressed barriers and enablers at the intra and interpersonal levels. For example, at the intrapersonal (physiotherapist) level, feedback from interviews indicated that this intervention was acceptable in enhancing the knowledge, skills and self-confidence of participants. This was likely due to specific components of the intervention that addressed these barriers, such as information provision at the continued education meeting and goal-setting, problem solving and feedback through the coaching process. Whereas, at the interpersonal (therapist-to-therapist) level, it seemed the barrier of lack of social support was not addressed adequately by the intervention components. Specifically, the process of encouragement and support through the continued education meeting did not enable support networks to be created within the primary care sites. Operationally, this intervention could also be further challenged by: (i) organisational barriers related to time and workload and (ii) patient expectations, which were originally identified in focus groups but deemed beyond the scope of this intervention. Indeed, both participants in the semi-structured interviews reported the ongoing negative effect of these barriers.

Consequently, future research should consider strategies to further enhance the intervention to address barriers beyond those at the physiotherapist level [39]. For example, participants in the focus groups highlighted the challenges of working with patients who expected passive rather than proactive treatment. This could perhaps be addressed by additional communication with referring primary care GPs about the scope of physiotherapy in chronic pain management as well as eliciting patient expectations and then managing them more effectively using a range of communication modes (e.g. introductory information advising patients what physiotherapy is and what they can expect prior to attending a consultation, this could be provided via postal, telephone or online methods).

Lastly, while we found that individualized coaching was acceptable and likely to provide a positive impact on behaviour; a one-to-one in person approach is costly and perhaps impedes opportunities for wider dissemination. Thus, exploration of providing this component in other formats and at different levels tailored to the individual could also be considered [40]. For example, the use of educational technology such as the virtual environment of Second Life [41] might be an alternative method for training HCPs and improving self-efficacy for the behaviour along with skill development. Additionally, the use of intermediaries such as, local opinion leaders [42] could be an alternative for targeting barriers related to professional role and identify and social influence. Both avenues may be promising and pragmatic ways to support clinician behaviour change.

## Conclusion

In conclusion, this study applied a systematic and rigorous process to the development of an intervention to support the implementation of an autonomy supportive communication style in primary care physiotherapy.  This process ensured that the intervention was theory-informed and evidence-based. Preliminary findings suggested that the intervention was both feasible and acceptable to recipients. These findings also support previous recommendations to adopt a multi-component approach and to consider context specific issues when implementing evidence based guidelines into clinical practice. 

## Abbreviations

LBP: Low back pain
CPG: Current clinical practice guidelines
HCPs: Health care professionals
SDT: Self-determination theory
KEDS: Knowledge exchange and delivery support
MRC: Medical Research Council
CONNECT: Communication style and exercise compliance in physiotherapy
TDF: Theoretical domains framework
BCT: Behaviour change techniques
HCCQ: Health care climate questionnaire
